# Characterization, Biological Activity, and Mechanism of Action of a Plant-Based Novel Antifungal Peptide, Cc-AFP1, Isolated From *Carum carvi*

**DOI:** 10.3389/fcimb.2021.743346

**Published:** 2021-09-29

**Authors:** Sima Sadat Seyedjavadi, Soghra Khani, Mehdi Goudarzi, Hadi Zare-Zardini, Masoomeh Shams-Ghahfarokhi, Fatemehsadat Jamzivar, Mehdi Razzaghi-Abyaneh

**Affiliations:** ^1^ Department of Mycology, Pasteur Institute of Iran, Tehran, Iran; ^2^ Department of Microbiology, School of Medicine, Shahid Beheshti University of Medical Sciences, Tehran, Iran; ^3^ Hematology and Oncology Research Center, Shahid Sadoughi University of Medical Sciences, Yazd, Iran; ^4^ Department of Biomedical Engineering, Meybod University, Meybod, Iran; ^5^ Department of Mycology, Faculty of Medical Sciences, Tarbiat Modares University, Tehran, Iran

**Keywords:** antifungal peptide, *Aspergillus fumigatus*, *Carum carvi*, cytotoxicity, mechanism of action, fungal infections, electron microscopy, drug discovery

## Abstract

Due to the increasing rate of invasive fungal infections and emerging antifungal resistance, development of novel antifungal drugs has been an urgent necessity. Antifungal peptides (AFPs) have recently attracted attention due to their unique ability to evade drug-resistant fungal pathogens. In this study, a novel AFP, Cc-AFP1, with a molecular weight of ~3.759 kDa, was isolated from *Carum carvi* L., purified by ammonium sulfate precipitation and reversed-phase HPLC and finally identified by sequence analysis using Edman degradation. Peptide sequence analysis revealed a fragment of 36 amino acid residues as RVCFRPVAPYLGVGVSGAVRDQIGVKLGSVYKGPRG for Cc-AFP1 with a net charge of +5 and a hydrophobicity ratio of 38%. The antifungal activity of Cc-AFP1 was confirmed against *Aspergillus* species with MIC values in the range of 8–16 µg/ml. Cc-AFP1 had less than 5% hemolytic activity at 8–16 µg/ml on human red blood cells with no obvious cytotoxicity against the HEK293 cell line. Stability analysis showed that the activity of Cc-AFP1 was maintained at different temperatures (20°C to 80°C) and pH (8 to 10). The results of a propidium iodide uptake and transmission electron microscopy showed that the antifungal activity of Cc-AFP1 could be attributed to alteration in the fungal cell membrane permeability. Taken together, these results indicate that Cc-AFP1 may be an attractive molecule to develop as a novel antifungal agent combating fungal infections cause by *Aspergillus* species.

## Introduction

In the past two decades, there has been an increase in the prevalence of life-threatening fungal infections leading to high morbidity and mortality (Firacative, 2020). Also, the increased rate of immunocompromised patients has led to the development of more serious fungal infections (Armstrong-James et al., 2014; Pathakumari et al., 2020). Over two million people are affected annually by invasive fungal diseases all over the world while fungal diseases cause more deaths every year compared with either tuberculosis or malaria (Bongomin et al., 2017). Global estimates of fungal diseases suggest a global annual occurrence of 300,000 cases of invasive aspergillosis and more than three million cases of chronic pulmonary aspergillosis (Banerjee et al., 2021). Invasive aspergillosis with more than 80% mortality rate is one of the most important nosocomial fungal infections caused mainly by *A. fumigatus*, followed by some other *Aspergillus* such as *A. flavus* as the second etiologic agent (De Cesare et al., 2020). Despite the increasing rate of fungal infections and the limited availability of effective antifungal drugs, only a few new antifungals have been developed into the market in recent years. Emerging antifungal drug resistance strengthens the need for novel antifungal molecule development as well (Chowdhary et al., 2017; Fernández de Ullivarri et al., 2020). Ideally, novel antifungal agents should have a broad-spectrum activity, being target-specific with multiple mechanisms of action without cross-resistance among fungal species. Among naturally occurring bioactive compounds with medicinal properties, antimicrobial peptides (AMPs) raised increasing attention due to their potent antimicrobial activity and simple structure (Cruz et al., 2014).

In this context, antifungal peptides (AFPs) which originated from natural sources such as plants and microorganisms gained increasing attention as therapeutic agents in recent years owing to several advantages, including high selectivity and effectiveness, low immunogenicity, and good penetration to host organs and tissues (Denning and Bromley, 2015). Antifungal peptides are diverse groups that are classified according to source, structure, and mode of action (Cruz et al., 2014; Fernández de Ullivarri et al., 2020). Natural AFPs are produced by many organisms, like vertebrate and invertebrate animals, plants, and microorganisms, and have a linear or cyclic structure with hydrophobic or amphipathic properties (Silva et al., 2014; Tam et al., 2015; Faruck et al., 2016). Based on the mechanism of action, antifungal peptides are divided into cell membrane- and intracellular-targeting peptides (Lécorché et al., 2012; Li et al., 2017).

The purification and identification of novel antifungal peptides have always been of interest in the current era of innovative therapies and management of human fungal pathogens (Meneguetti et al., 2017). Plants produce a large variety of AFPs that have the potential to be used in the development of novel antifungal therapeutics (Campos et al., 2018). These peptides are cysteine-rich and commonly found in seeds and, based on amino acid sequence homology, comprise a number of classes (De Lucca, 2000).

*Carum carvi* L. (Caraway) is an annual herbaceous plant that belongs to the Umbelliferae family. The plant is native to some certain regions of Iran and is used in foods and herbal medicine (Razzaghi-Abyaneh et al., 2009; Panda, 2010; Nasiri et al., 2020). To our knowledge, very little has been documented about AFPs that originated from *C. carvi*. In the present study, we isolated and characterized a novel AFP from *C. carvi* seeds with an α-helix and random coil structure which showed strong antifungal activity against human and animal pathogenic *Aspergillus* species, while it has no obvious cytotoxicity against human red blood cells and the HEK293 cell line *in vitro*.

## Materials and Methods

### Fungal Strain and *Carum carvi*

Seeds of *Carum carvi* were prepared from the herbarium of the Research Institute of Forests and Rangelands, Tehran, Iran. They were washed with clean water and then were dried at room temperature. *Aspergillus* species including *A. fumigatus* PFCC 50091, *A. niger* ATCC 9142, and *A. flavus* PFCC 50041 were provided by the Pathogenic Fungi Culture Collection of the Pasteur Institute of Iran (http://fa.pasteur.ac.ir/VisitDetails.aspx?Id=1311). The strains were kept in Sabouraud dextrose broth (SDB, Merck, Germany) including 20% glycerol at -70°C. The strains were subcultured in Sabouraud dextrose agar (SDA, Merck, Germany).

### Peptide Extraction and Purification

In brief, *Carum carvi* seeds were ground to a fine flour, and total protein was extracted with an extraction buffer (100 mM KCl, 15 mM NaH_2_PO_4_, 10 mM Na_2_HPO_4_, and 1.5% EDTA) pH 5.4, in the shaker at 4°C for 4 h (Games et al., 2008). Subsequently, the supernatant was filtered (Whatman No 1, pore size 11 μm) and saturated with 85% ammonium sulfate ((NH_4_)_2_SO_4_ for 24 h at 4°C. The precipitate, formed overnight, was extensively dialyzed against distilled water by using benzoylated membrane performance (MWCO 2,000 Da) (Sigma Aldrich-USA) at 4°C within 12 h to remove the residual (NH_4_)_2_SO_4_. For isolating low molecular weight peptides, the protein extract was filtered through Amicon Ultra 15-ml 10,000 MWCO centrifugal filters (Millipore, USA) followed by lyophilization. The reversed-phase HPLC column (C18 column, 7.8 × 300 mm; Tosoh, Tokyo, Japan) with the gradient of 5%–65% (v/v) solution B (0.098% TFA in acetonitrile) and A (0.1% TFA in water) at a flow rate of 1 ml/min for 85 min was employed for purifying the antifungal peptides from lyophilized extracts (Asoodeh et al., 2012). According to the absorbance at 220 nm, the peaks were collected and then lyophilized for determining the antifungal effect and those that had the highest activity were collected. For checking the peak with antifungal activity purity, this peak was re-chromatographed in the same column using the similar solvent system in the same conditions.

### Tricine–SDS-PAGE

For evaluating the molecular weight as well as the purity of the peak with antifungal activity, we performed the tricine–sodium dodecyl sulfate-polyacrylamide gel electrophoresis (Tricine–SDS-PAGE) under reducing conditions on a Bio-Rad electrophoresis apparatus based on the Schagger approach (Schägger and Von Jagow, 1987). Samples were mixed with 0.5 ml of sample buffer (0.05 M Tris–HCl, 4% SDS, 12% glycerol, 0.01% bromophenol blue, and 2% 2-ME) and boiled for 10 min. Then, samples were run on a 16.5% Tris-tricine gel with tricine–SDS running buffer for overnight in 25 V tension. Silver staining was used to stain the gel. A protein ladder (2–250 kDa) was used as a standard for determining the proximate molecular mass.

### Radial Diffusion Assay

The antifungal activity of the collected peaks was assessed using a radial diffusion assay (RDA) according to the Wang approach (Wang et al., 2015). The *Aspergillus* species spore suspension was obtained by scraping the culture surface slightly by a sterile glass rod following the addition of enough 0.1% aqueous solution of Tween 80. The suspension was passed through sterile cheesecloth to remove mycelia fragments. Briefly, fungal cells were added to Sabouraud dextrose agar fixed at 42°C followed by a quick dispensing into a Petri dish. The wells were punched, and 10 µl of the peptide from the stock of 200 µg/ml in the final concentration of 2 µg/well was loaded in to the wells. Amphotericin B (0.2 μg/well) was considered as a positive control. Following incubation at 35°C for 48 h, the clear zones around the wells were assessed. All the experiments were performed twice in triplicates.

### Minimum Inhibitory Concentrations and Minimum Fungicidal Concentrations

The minimum inhibition concentration assay (MIC) of the purified and active peptide was measured as described by Li et al. (Li et al., 2015) after some modifications. In brief, the assay was determined by an inoculum of 10^5^ conidia/ml in Sabouraud dextrose broth and evaluated in 96-well microplates. Serial two-fold dilutions were provided for obtaining final levels (1 to 250 μg/ml). Amphotericin B (0.016 to 4 μg/ml) was used as control. Incubation of the plates was done (35°C/48 h). The minimum inhibitory concentration (MIC) was defined as 99% inhibition of fungal growth in 96-well microplates by visual assay. Twenty microliters of the specimens of each well was then isolated and plated on Sabouraud dextrose agar plates. The plates were incubated at 35°C for 48 h. The minimum fungicidal concentration (MFC) was defined as the lowest peptide level to kill fungal cells.

### Mass Spectrometry and N−Terminal Sequence Analysis

The molecular mass of the isolated antifungal peptide was determined by electrospray ionization mass spectrometry (MS) at a mass to charge (m/z). The amino acid sequence of the purified antifungal peptide was determined using Edman degradation. Accordingly, an ABI Procise Edman Micro Sequencer (Model 492) was connected online to the 140C ABI PTH Amino Acid Analyzer.

### Sequence Alignment and Phylogenetic Tree

The search for similar sequences was performed by database search (for finding similar peptides with the highest similarity to the new peptide) and CLC main workbench software. In this section, 10 peptides with the highest similarity to the new peptide were obtained after database search. Program blast was done for comparison of the sequences of these peptides with the new peptide. Alignment was manually adjusted, and a phylogenetic tree was acquired by CLC Main Workbench software. For evaluation of reproducibility of the tree topology, the phylogenetic tree was measured using bootstrap analysis with 100 replications.

### Bioinformatics Analysis

For general AMP prediction, the APD3 prediction server (https://wangapd3.com/main.php) was used. The AMP probability of the peptide was anticipated through machine learning algorithms support vector machine (SVM), artificial neural network, random forest, and discriminant analysis using the CAMP_R3_ server (http://http://www.camp3.bicnirrh.res). Each algorithm threshold was from 0.5 to 1. Peptides with the threshold number of >0.5 were AMP. The physicochemical characteristics of the antifungal peptide were predicted using the ExPASy Proteomics server (http://www.expasy.org/tools/protparam.html) for the net charge, hydrophobic ratio, Boman index, isoelectric point (pI), values of the instability index, aliphatic index, and molecular weight. To predict the antifungal activity of the expected peptide, the online server iAMPpred (http://cabgrid.res.in:8080/amppred/server.php) was used. Helical wheel projection was done for predicting the amino acid position in peptides using the HeliQuest server (https://heliquest.ipmc.cnrs.fr/cgi-bin/ComputPara ms.py). The three-dimensional structure of the peptide was estimated online by the I-TASSER server (http://zhanglab.ccmb.med.umich.edu/ITASSER/). The model quality was measured through Accelrys, DS Visualizer ver. 1.7.

### Peptide Synthesis

The peptide was chemically synthesized by Biomatik Co. (Ontario, Canada) using 9-fluorenylmethoxycarbonyl (F-moc) solid-phase chemistry (Merrifield, 1986). Peptide purity and mass identity were confirmed by reversed-phase high-performance liquid chromatography (RP-HPLC) and electrospray ionization–mass spectrometry (ESI-MS, Waters ZQ 2000, Milford, MA, USA), respectively.

### Hemolytic Activity

The peptide hemolytic activity was calculated as defined by Wu et al. (2014). Fresh heparinized human whole blood was centrifuged (5 min/4°C). The achieved erythrocytes were washed three times followed by resuspending in PBS. Then, the peptide serial dilution (1–128 µg/ml) was mixed with erythrocytes and subjected to incubation (1 h/37°C). Erythrocytes after treatment with PBS were applied as a negative control and 0.1% Triton X-100 as positive control. Hemoglobin release was measured through the measurement of the absorption at 567 nm using the ELISA reader. Hemolysis (%) = [test OD − negative control OD)/(positive control OD – negative control OD)] × 100.

### Cytotoxicity Assays

The cytotoxic effects of the antifungal peptide on human embryonic kidney cell line 293 (HEK293) was calculated by 3-(4,5-dimethylthiazol-2-yl)-2,5-diphenyltetrazolium bromide (MTT) assays (Mosmann, 1983). In brief, Dulbecco’s modified Eagle’s medium (DMEM) treated with 10% (v/v) fetal bovine serum (FBS) was used for the growth of the cell lines and was subjected to incubation with 5% CO_2_ at 37°C. Seeding the cells was done by plates of 96 wells with about 5,000 cells per well. Following overnight culture at 37°C in 5% CO_2_, different peptide levels (1–128 µg/ml) were used for the treatment of the cells for 24 h. Next, incubation was done using 50 ml of MTT (0.5 mg/ml) for 4 h. Following incubation, the plates were centrifuged (5 min), and the supernatants were removed, and for dissolving the formazan crystals, DMSO (dimethyl sulfoxide; 150 ml) was added. At the end, the ELISA reader was used to measure the absorbance at 570 nm. The findings were obtained from three separate experiments, each conducted in triplicate.

### Temperature and pH Stability Assay

The peptide stability at different temperatures and pH values was calculated as defined by Batdorj et al. (2006). Heat sensitivity was evaluated after incubation at various temperatures (10°C–100°C) for 1 h. The peptide without heating at various temperatures was considered as a control. For pH stability testing, the samples were adjusted to 2–10 with 1 mol/l HCl or 1 mol/l NaOH and placed at 25°C for 1 h. Then, the pH value was fixed at 7.0 before antifungal assays. The peptide dissolving in solution (pH: 7.2) was considered as a control. The tests were performed three times. *A. fumigatus* was applied as an indicator for detecting the antifungal activity that was assessed using radial diffusion assay.

### Determination of Fungal Cell Integrity Using PI Uptake

To determine the membrane permeability of the antifungal peptide on *A. fumigatus* hyphae, the propidium iodide (PI) uptake assay was used according to the fluorescence microscopy assay.

In brief, a conidia suspension of *A. fumigatus* (10^6^ conidia/ml) in SDB was poured on a six-well microplate and incubated (35°C, 24 h). Next, the hyphae were incubated with the antifungal peptide at the concentrations of 8 and 16 µg/ml for 4 h at 35°C *via* constant shaking (120 rpm). Subsequently, the PI solution with a final concentration of 50 μg/ml was added to each well for 15 min at room temperature in the dark. Next, the stained hyphae specimens were visualized by fluorescence microscopy (Eclipse 80i, Nikon, Japan) with appropriate filters (excitation/emission at 530/590 nm). Untreated fungal hypha was used as negative controls.

### Transmission Electron Microscopy

TEM was performed as described previously (Bozzola and Russell, 1999) with some modifications. For evaluating the peptide effect on hyphae morphology, we cultured the conidia suspension of *A. fumigatus* (10^6^ conidia/ml) in SDB (1 ml) in microplates of 24 wells (24 h/35°C) followed by exposure to antifungal peptide at the concentrations of 16 and 32 µg/ml and incubation at 35°C for 4 h (Ries et al., 2017). Then, the specimens were fixed using 2.5% (v/v) glutaraldehyde (3 h/4°C). Following three times washing in 0.1% PBS, the samples were then fixed using 1% osmium tetroxide in PBS within 70 min and were subjected to washing for two times with PBS dehydrated in gently increasing acetone solutions, and embedding in Epon 812. The ultra-microtome was used to cut ultrathin sections, and staining with uranyl acetate and lead citrate was done and observed by a Zeiss EM 900 TEM device at 80 kV. The non-treated hypha was considered as control.

### Statistical Analysis

Values were expressed as mean ± SD. Statistical analysis was done with GraphPad Prism 5 Statistical software (GraphPad Software, Inc., La Jolla, CA, USA).

## Results

### Peptide Purification and Tricine–SDS-PAGE

**Figure 1:**
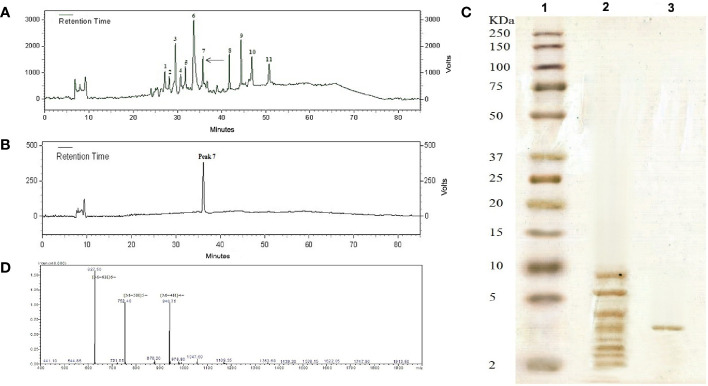
**(A)** Peptide RP-HPLC purification from *Carum carvi* seeds. The arrow shows the active peak 7. **(B)** The chromatogram obtained after the re-chromatography process to purify the active peak 7. **(C)** Tricine–SDS-PAGE profile stained with silver (1) molecular weight markers (2–250 kDa), (2) the extracted peptide passing through an ultra-membrane with the cutoff of 10 kDa, and (3) the RP-HPLC column purified peak 7. **(D)** ESI- mass spectrum of purified antifungal peptide. Multiple charged molecular ions are indicated.

The low molecular weight peptides (less than 10 kDa) obtained from *Carum carvi* seeds were lyophilized and subjected to reversed-phase chromatography, and after fractionation using a C18 column, 11 fractions were obtained (**Figure 1A**). According to the increase and decline of the absorbance of the peaks at 220 nm, each peak was manually collected and 11 peaks were obtained from the C18 RP-HPLC as an individual peak to be tested for the antifungal activity against *Aspergillus* species. Out of 11 peaks, Peak 7 which showed the strongest antifungal activity was chosen for further studies. For more purification, this active peak with antifungal activity was applied again to the same column for re-chromatography under the same elution conditions (**Figure 1B**). A single peak was observed by monitoring the eluted fraction at 220 nm (**Figure 1B**). This peak was further analyzed by SDS-PAGE analysis, mass spectrometry, and N terminal sequencing. **Figure 1C** indicates the electrophoresis pattern of the extracted peptides passed through an ultra-membrane with a 10-kDa cutoff and active peak with antifungal activity purified from the RP-HPLC column on Tricine–SDS-PAGE. Following the purification processes as well as SDS-PAGE analysis, a single band with a molecular weight of about 3.7 kDa was obtained.

### Mass Spectrometry and Amino Acid Sequence

Because of its antifungal activity, the peptide band of peak 7 (~3.7 kDa band) was chosen for further characterization. ESI mass analyses of the purified peptide showed that the molecular mass of this peptide was 3,759.43 Da (**Figure 1D**). The result was in close agreement with the relative molecular weight indicated by Tricine–SDS-PAGE. To determine the amino acid sequence, the peptide was sequenced *via* Edman degradation. From this analysis, a fragment of 36 amino acid residues was obtained as RVCFRPVAPYLGVGVSGAVRDQIGVKLGSVYKGPRG.

### Antifungal Activity

**Figure 2:**
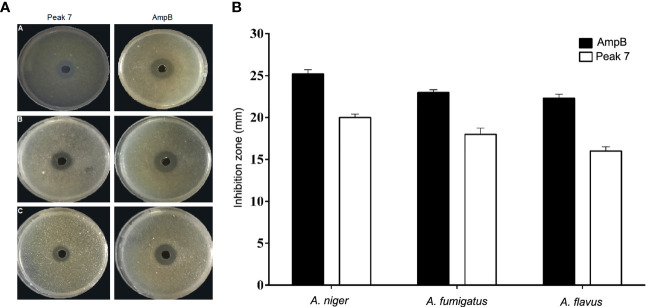
**(A, B)** Antifungal effect of peak 7 against *Aspergillus* species was measured using the radial diffusion method. **(A)** The inhibition zone of peak 7 (2 μg/well) and amphotericin B (0.2 μg/well) against (A) *A. flavus* PFCC 50041, (B) *A. niger* ATCC 9142, and (C) *A. fumigatus* PFCC 50091. **(B)** Quantitative results of the inhibition zone diameters of peak 7 and AmpB for tested fungi with values that are given as the mean ± SD.

Analysis of the biological activity of 11 peaks purified by HPLC using RDA showed only three peaks of antifungal activity against *Aspergillus* species. The inhibition zone diameters for two peaks, i.e., 3 and 5, were reported around 5 mm, which showed a very weak negligibility against the tested fungi. Peak 7 showed strong antifungal activity against all *Aspergillus* species and was considered as the main bioactive peptide of *C. carvi* seeds. The inhibition zones of peak 7 and AmpB as a common antifungal compound against *Aspergillus* species are shown in **Figure 2A**. The diameters of the inhibition zones of *Aspergillus* species were 16–20 mm for peak 7, while they were 22–25 mm for AmpB (**Figure 2B**). The peptide antifungal activity was assessed further as MIC values by the micro broth dilution method. As shown in **Table 1**, the peptide was active against *Aspergillus* species, with MIC values that ranged 8–16 µg/ml. Amphotericin B showed the MIC range of 0.25–0.5 µg/ml, and the MFCs of the peptide against the *Aspergillus* species are equal or two times higher than its MICs.

**Table 1:** MIC and MFC values (μg/mL) of Cc-AFP1 and amphotericin B (AmpB) for *Aspergillus* species.

Fungal strain	MIC (μg/mL)		MFC (μg/mL)	
	Cc-AFP1	AmpB	Cc-AFP1	AmpB
*Aspergillus fumigatus* PFCC 50091	8	0.5	16	0.5
*Aspergillus flavus* PFCC 50041	16	0.5	32	1
*Aspergillus niger* ATCC 9142	8	0.25	8	0.5

### Sequence Alignment and Phylogenetic Tree

**Figure 3:**
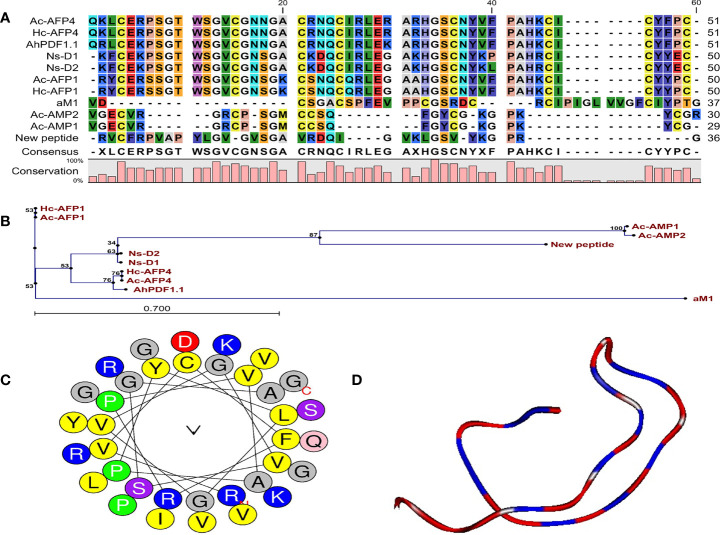
Characteristic features and structural data of Cc-AFP1. **(A)** The alignment of Cc-AFP1 amino acid sequences as well as the sequences related to other antimicrobial peptides. **(B)** Phylogenetic tree of Cc-AFP1. Phylogenetic tree was acquired by CLC main workbench software. Each sequence name is written at the end of the relevant branch. The tree reliability was determined using bootstrap method with 100 replications. **(C)** Helical wheel diagram of Cc-AFP1 peptide and polar and non-polar amino acids, and their locations in peptide can be observed. **(D)** Three-dimensional structure model of The Cc-AFP1 with random coil.

The obtained peptide sequence indicated no full sequence homology to the reported AMPs, and BLAST results further confirmed that this peptide was a novel antifungal peptide from *Carum carvi* seeds. Sequence alignment and phylogenetic tree showed that the new peptide has the highest sequence similarity (36%) with Ns-D2 purified from seeds of *Nigella sativa* L. (**Figures 3A, B** **)** (Rogozhin et al., 2011). Based on this similarity and source of peptide purification (*Carum carvi*), this new peptide was named Cc-AFP1.

### Bioinformatics and Structural Analysis

Generally, the predictions of online servers of CAMP_R3_ (**Table 2**) and iAMPpred indicated that Cc-AFP1 is AMP and has antifungal activity. **Table 2** presents the sequences and key physicochemical characteristics of Cc-AFP1. Its net charge, hydrophobic residue, protein-binding potential (Boman index), and pI were equal to +5, 38%, 0.96, and 10.44, respectively. The values of the instability index and aliphatic index for this peptide were 8.99 and 94.44, respectively. The helical wheel diagram of the Cc-AFP1 peptide showed that this peptide has a secondary amphipathic conformation and possessed a hydrophobic face (**Figure 3C**). The tertiary structure prediction using the server I-TASSER showed a random coil structure in the Cc-AFP1 peptide (**Figure 3D**). The C-score can be used to estimate the quality of anticipated models using I-TASSER (in the range of −5.0 to 2.0). The considered C-score of Cc-AFP1 was -2.07, which indicated the model correct global topology.

**Table 2:** The probability of antimicrobial activity and physicochemical features of Cc-AFP1.

Sequence	% hydrophobic	Net charge	Boman index	Molecular weight (Da)	Instability index	Aliphatic index	Score of algorithms		
							SVM	RF	ANN
RVCFRPVATYLGVCGVSGACRDRCVKLGSCVYKGPG	38	5	0.96	3759.43	8.99	94.44	0.96	0.98	AMP

### Hemolytic Activity and Cytotoxicity

**Figure 4:**
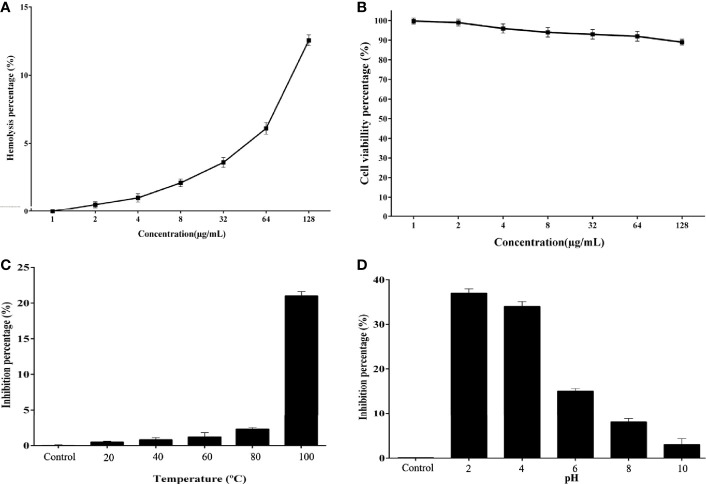
Hemolytic effect, cytotoxic effect, and stability of Cc-AFP1. **(A)** Cc-AFP1 hemolytic effect against human erythrocytes. **(B)** Cytotoxic effect of Cc-AFP1 against HEK293 cells. **(C, D)** The temperature **(C)** and pH **(D)** effects on the antifungal effect of Cc-AFP1 against *A. fumigatus* cells. Values are provided as mean ± SD.

The Cc-AFP1 hemolytic activity was measured in human erythrocytes. As shown in **Figure 4A**, the Cc-AFP1 toxicity at its MIC (8–16 μg/ml) and MFC (16-32 μg/ml) ranges was less than 5%. Cc-AFP1 induced 12.56% of hemolytic activity at the highest tested concentration (128 μg/ml). The toxicity of the Cc-AFP1 against HEK293 cells was assessed by serial peptide levels ranging between 2 and 128 μg/ml (**Figure 4B**). Cc-AFP1 had a low (>6) toxicity against HEK293 cells at 8–32 μg/ml.

### Effects of Temperature and pH on Antifungal Activity

We measured the effect of temperature on the Cc-AFP1 (**Figure 4C**), and based on the findings, Cc-AFP1 was stable at various temperatures (20°C to 80°C) for 1 h. Interestingly, a low reduction of 21.6% for *A. fumigatus* was observed at 100°C during 1 h of treatment.

**Figure 4D** indicates the pH effects on the antifungal peptide stability. Cc-AFP1 remained stable at pH values ranging from 8 to 10. However, its activity was reduced at pH 2, 4, and 6.0.

### Effects of the Peptide on Cell Membrane Permeability

**Figure 5:**
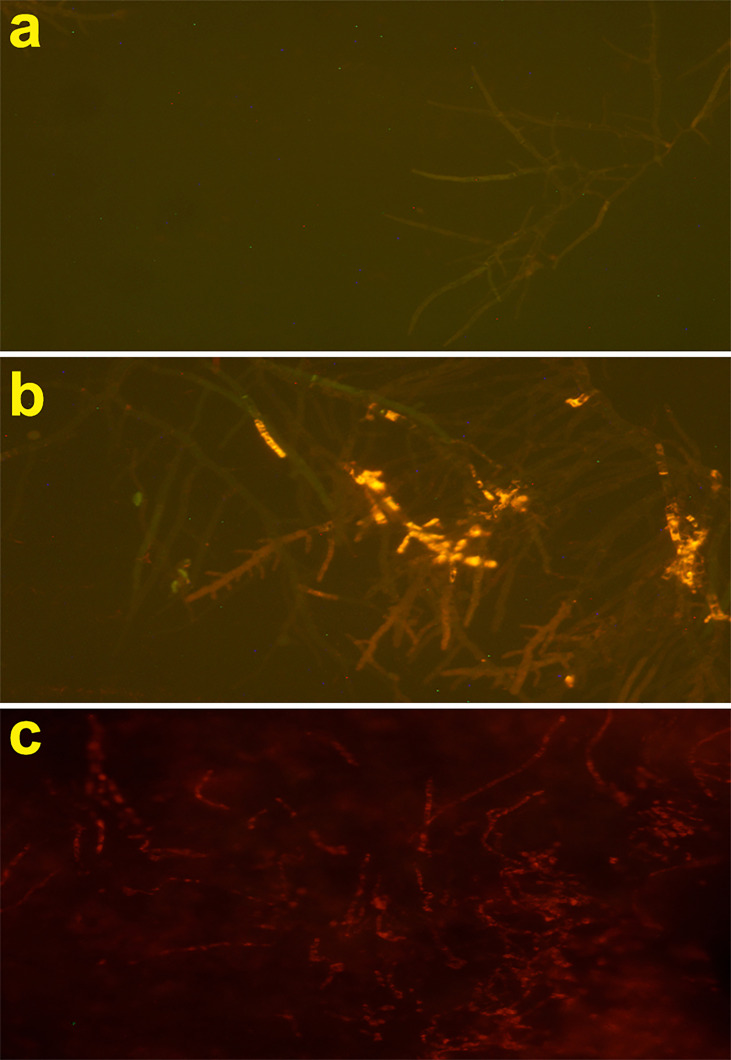
Fluorescent microscope analysis of propidium iodide uptake during the membrane permeabilization assay. **(A)** Fluorescent images of untreated *A. fumigatus* hyphae. **(B, C)** Fluorescent images of treated *A. fumigatus* hyphae with Cc-AFP1 at the concentration of 8 and 16 μg/ml, respectively.

The effect of Cc-AFP1 on the integrity of the hypha membrane of *A. fumigatus* was investigated using the PI uptake procedure by fluorescence microscopy. PI (a DNA-staining fluorescent probe) could enter the cell when the cell membrane is damaged and binds with nucleic acids, producing a red fluorescence. As shown in **Figure 5**, the fluorescent intensity increased in the hyphae of *A. fumigatus* when the Cc-AFP1 concentration was increased from 8 to 16 µg/ml; however, there was no fluorescence in the control. The result of the PI uptake indicated that Cc-AFP1 could disrupt the integrity of the cell membrane.

### Morphological Observation

**Figure 6:**
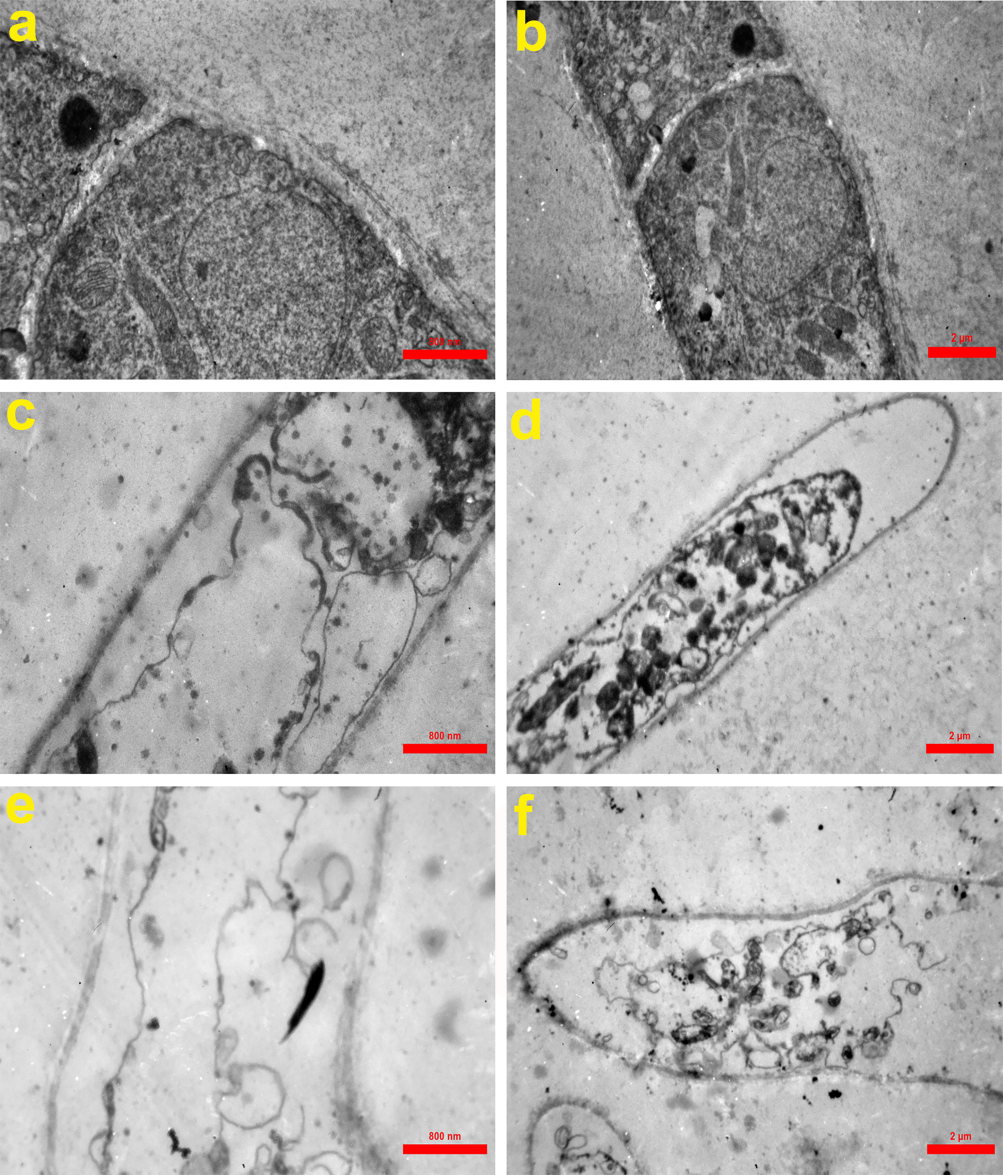
Transmission electron microscopy of *A. fumigatus*-untreated hyphae **(A, B)** and hyphae exposed to Cc-AFP1 at the concentrations of 16 µg/ml **(C, D)** and 32 µg/ml **(E, F)**. Untreated hyphae of *A. fumigatus* had intact cell walls, integrated cell membranes, and normal organelles **(A, B)**. In contrast, in treated hyphae with Cc-AFP1, pathologic ultrastructure changes including attenuated irregular cell wall, loss of the structural integrity and shrinkage of the cell membrane, detachment of the cell membrane from the cell wall, cytoplasmic depletion, and membranous damage of cytoplasmic organelles especially mitochondria were evident **(C–F)**.

According to **Figure 6**, untreated hypha of *A. fumigatus* had intact cell walls, integrated cell membranes, and normal organelles (**Figures 6A, B** **)**. In contrast, in treated hyphae with Cc-AFP1 at the concentration of 16 µg/ml, pathologic ultrastructure changes including attenuated irregular cell wall, loss of the structural integrity and shrinkage of the cell membrane, detachment of the cell membrane from the cell wall, cytoplasmic depletion, and membranous damage of cytoplasmic organelles especially mitochondria were evident (**Figures 6C, D** **)**. Massive destruction of cell membranes and cytoplasmic organelles together with whole depletion of hyphae content were observed in the fungus exposed to 32 µg/ml concentration of the peptide (**Figures 6E, F** **)**.

## Discussion

The diversity of AMPs in plants is large, and so far hundreds of different peptides have been characterized. The present work focused on the isolation, purification, characterization, and mechanisms *in vitro* of a novel antifungal peptide, Cc-AFP1, in the protein extract of *Carum carvi* seeds. According to the BLAST results, Cc-AFP1 showed no complete sequence homology to any reported AMPs from AMP databases, which strongly indicates that it is a new plant antifungal peptide from *Carum carvi seeds*. In our research, the *in silico* predictive and physicochemical properties of Cc-AFP1 were investigated by several online tools before proceeding with the *in vitro* experiments. Cc-AFP1 consists of 36 amino acids in total with a net charge of +5, which is important for the electrostatic interaction with the negative charges of fungal membrane components (Kim et al., 2014).

Cc-AFP1 also contains seven hydrophobic residues with a total hydrophobic ratio of 38%, which may be involved in the disruption of fungal cell membrane integrity. Studies showed that hydrophobicity is one of the important parameters of AMP activity (Chen et al., 2007; Kim et al., 2014). Hydrophobic residues are needed to interact with the lipid bilayer to create pores in the fungal cell cytoplasmic membrane. Finally, such interactions degrade the fungal cytoplasmic membrane where the non-polar face of the AMP can be inserted into the membrane by hydrophobic interactions (Chen et al., 2007). The Boman index of Cc-AFP1 is 0.96 kcal/mol, and the positive Boman index can increase the Cc-AFP1 capacity to attach to fungal cell membrane proteins (He et al., 2018). This index can estimate the protein ability to attach to another protein (Boman, 2003). The instability index is 8.99; this value indicates the stability of the peptide in a test tube which should be less than 40. Furthermore, the aliphatic index is 94.44 which is linked to thermostability and is considered as the relative volume of side chains (Ala, Val, Ile and Leu) (Guruprasad et al., 1990).

Structure prediction by helical wheel projection revealed that some hydrophobic residues, such as valine and leucine, accumulated in one side of the structure, and this may lead to facilitate the insertion of the non-polar face of the antifungal peptide into the membrane *via* hydrophobic interactions. On the other hand, positive polar amino acids on the structure can support phospholipid binding after the attachment of AMP to the cell membrane (He et al., 2018).

According to the sequence alignment and phylogenetic tree results, Cc-AFP1 showed the highest sequence similarity (36%) with the Ns-D2 antifungal peptide from the plant source. Ns-D2 was isolated from Nigella sativa L. seeds and showed an effective antifungal activity against fungi such as *A. niger*, *Fusarium* species, and *Botrytis cinerea*. The net charge and percentage of hydrophobic residue of Ns-D2 were +3 and 36%, respectively (Rogozhin et al., 2011).

The prediction results and physiochemical properties of Cc-AFP1 make it a promising AFP candidate. In our studies, we also confirmed the *in silico* results experimentally.

In this paper, MICs and MFCs of Cc-AFP1 have been measured against *Aspergillus* species and Cc-AFP1 showed a remarkable inhibitory effect against *Aspergillus* species with the MICs comparable with other antimicrobial peptides.

Antifungal peptides act *via* different mechanisms, like increasing permeability and disruption of fungal membranes, reactive oxygen species generation and apoptosis, or intracellular receptor binding (Van der Weerden et al., 2013). In this study, Cc-AFP1 increased the membrane permeability, which was confirmed by PI uptake assay. Only dead fungal cells with damaged cell membranes can take up PI dye, and *A. fumigatus* treated with Cc-AFP1 was able to take up more PI dye than the control. Many studies have confirmed the cell membrane damage after treatment with AMPs by the PI uptake assay (Dathe et al., 2002; Li et al., 2016; Lyu et al., 2016). TEM results also demonstrated the induction of membrane damage and loss of cell surface integrity by Cc-AFP1. These results are comparable to many other studies that used TEM to determine the damage present in the fungal cell membrane after treatment with AMP (Lyu et al., 2016; Do Nascimento Dias et al., 2020).

Overall, these observations and evidence indicate that membrane damage and permeabilization are one of the mechanisms of action of this new peptide that has been previously reported for many antimicrobial peptides (Maurya et al., 2011). However, it is hard to recognize the mode of action of Cc-AFP1 and more studies should be done to better understand the exact targets of this peptide.

The AMPs’ therapeutic effects are associated with the AMPs’ cell selectivity, by which they destroy pathogens with no obvious cytotoxic effect to mammalian cells. Cell selectivity can evaluate the AMPs’ capacity to differentiate pathogens from host cells. As shown in **Figure 4A**, Cc-AFP1 indicated a negligible (>5%) hemolytic activity against the studied RBCs in effective concentrations over MIC and MFC ranges. At higher concentrations, some meaningless degrees of cytotoxic effects and hemolytic activity were reported for Cc-AFP1. The hemolytic activity against human RBC is correlated with peptide hydrophobicity, and previous studies showed that peptides characterized by high hydrophobicity have a tendency to penetrate deeply into the red blood cell membrane hydrophobic core and perform hemolytic activities (Jiang et al., 2008).

Besides cell selectivity of the AMPs, their stability is an important challenge to use them for therapeutic purposes. Adding co-solvents to peptide solutions has different effects, like denaturation or reduced activity. Surprisingly, the Cc-AFP1 antifungal activity was not obviously changed under a wide range of temperatures and pH. The thermal stability of Cc-AFP1 can be due to the nature and chemical structure of the peptide demonstrated by aliphatic indices, calculated by the APD databases.

## Conclusion

In the present study, Cc-AFP1 as a novel antifungal peptide with α-helix and random coil structure was isolated from *Carum carvi* seeds and characterized through reversed-phase HPLC and Edman degradation methods. The peptide showed a remarkable antifungal activity against human pathogenic *Aspergillus* species, while it had no obvious hemolytic activity and cytotoxicity *in vitro*. Cc-AFP1 was shown to damage the fungal cell wall and cell membrane, increase cell permeability, and disrupt membranous structures of the fungal cells. Taken together, these results indicate that Cc-AFP1 may be an attractive molecule to develop as a novel antifungal agent combating fungal infections caused by *Aspergillus* species. Further works to investigate spectrum of antifungal activity, the exact mechanism of action and structure–activity relationship of Cc-AFP1 are recommended.

## Data Availability Statement

The original contributions presented in the study are included in the article/supplementary material. Further inquiries can be directed to the corresponding author.

## Ethics Statement

This study was approved by the Ethics Committee of the National Institute for Medical Research Development (NIMAD) under ethics number IR.NIMAD.REC.1396.121.

## Author Contributions

SS, and MR-A designed and conceived of the study. SS, SK, HZ-Z, MG, MS-G, FJ, and MR-A carried out the experimental work. SS, MG, and MR-A wrote the paper. All authors contributed to the article and approved the submitted version. MR-A supervised the study.

## Funding

This work was supported by the Elite Researcher Grant Committee under award numbers 958634 and 963646 from the National Institute for Medical Research Development (NIMAD), Tehran, Iran. to MR-A.

## Conflict of Interest

The authors declare that the research was conducted in the absence of any commercial or financial relationships that could be construed as a potential conflict of interest.

## Publisher’s Note

All claims expressed in this article are solely those of the authors and do not necessarily represent those of their affiliated organizations, or those of the publisher, the editors and the reviewers. Any product that may be evaluated in this article, or claim that may be made by its manufacturer, is not guaranteed or endorsed by the publisher.
